# The use of microbubbles to target drug delivery

**DOI:** 10.1186/1476-7120-2-23

**Published:** 2004-11-16

**Authors:** Jeane M Tsutsui, Feng Xie, Richard Thomas Porter

**Affiliations:** 1 Department of Internal Medicine, Section of Cardiology, University of Nebraska Medical Center, Omaha, Nebraska, USA

## Abstract

Ultrasound-mediated microbubbles destruction has been proposed as an innovative method for noninvasive delivering of drugs and genes to different tissues. Microbubbles are used to carry a drug or gene until a specific area of interest is reached, and then ultrasound is used to burst the microbubbles, causing site-specific delivery of the bioactive materials. Furthermore, the ability of albumin-coated microbubbles to adhere to vascular regions with glycocalix damage or endothelial dysfunction is another possible mechanism to deliver drugs even in the absence of ultrasound. This review focuses on the characteristics of microbubbles that give them therapeutic properties and some important aspects of ultrasound parameters that are known to influence microbubble-mediated drug delivery. In addition, current studies involving this novel therapeutical application of microbubbles will be discussed.

## Introduction

The recent advances in gene therapy and molecular biology have improved the interest in methods of noninvasive delivery of therapeutic agents. Besides the well known application of microbubbles as contrast agents for diagnostic ultrasound, microbubbles have also been demonstrated an effective technique for targeted delivery of drugs and genes [1-6]. Drugs can be incorporated into the microbubbles in a number of different ways, including binding of the drug to the microbubble shell and attachment of site-specific ligands. As perfluorocarbon-filled microbubbles are sufficiently stable for circulating in the vasculature as blood pool agents, they act as carriers of these agents until the site of interest is reached. Ultrasound applied over the skin surface can then be used to burst the microbubbles at this site, causing localized release of the drug [7-10]. This technique then permits using lower concentrations of drugs systemically, and concentration of the drug only where it is needed. This improved therapeutic index may be extremely advantageous in cases of drugs with hazardous systemic side effects, like cytotoxic agents. Albumin-encapsulated microbubbles have also demonstrated to adhere to the vessel walls in the setting of endothelial dysfunction [11]. This also may be a method of targeting delivery with microbubbles but without the application of ultrasound.

### Mechanisms for Target Drug Delivery Using Microbubbles

Two possible strategies for delivering drugs and genes with microbubbles are emerging. The first consists on the ultrasound-mediated microbubble destruction, which is based on the cavitation of microbubbles induced by ultrasound application, and the second is the direct delivery of substances bound to microbubbles in the absence of ultrasound. Different drugs and genes can be incorporated into the ultrasound contrast agents. It has already been demonstrated that perfluorocarbon-filled albumin microbubbles avidly bind proteins and synthetic oligonucleotides [12]. In a similar way, microbubbles can directly take up genetic material, such as plasmids and adenovirus [12,13], and phospholipid-coated microbubbles have a high affinity for chemotherapeutic drugs [14]. Furthermore, specific ligands for endothelial cell adhesion molecules, such as P-selectin and leukocyte intercellular adhesion molecule 1 (ICAM-1), can be attached to both lipid- and albumin-encapsulated microbubbles, which increases their deposition to activated endothelium [15,16].

The mechanisms by which ultrasound facilitates the delivery of drugs and genes result from a complex interplay among the therapeutic agent, the microbubble characteristics, the target tissue, and the nature of ultrasound energy. The presence of microbubbles in the insonified field reduces the peak negative pressure needed to enhance drug delivery with ultrasound. This occurs because the microbubbles act as nuclei for cavitation, decreasing the threshold of ultrasound energy necessary to cause this phenomenon. The results of optical and acoustical studies have suggested the following mechanisms for microbubble destruction by ultrasound: 1- gradual diffusion of gas at low acoustic power, 2- formation of a shell defect with diffusion of gas, 3- immediate expulsion of the microbubble shell at high acoustic power, and 4- dispersion of the microbubble into several smaller bubbles. Cavitation of the bubbles is characterized by rapid destruction of contrast agents due to a hydrodynamic instability excited during large amplitude oscillations, and is directly dependent on the transmission pressure [17,18]. It has been reported that the application of ultrasound to contrast agents creates extravasation points in skeletal muscle capillaries [2,19], and this phenomenon is dependent on the applied ultrasound power. High intensity ultrasound (referred to as a high mechanical index) can rupture capillary vessels, resulting in deposit of protein and genetic material into the tissues. Skyba et al [1] demonstrated in an exteriorized spinotrapezius preparation that ultrasonic destruction of gas-filled microbubbles caused rupture of microvessels with diameter ≤ 7 μm (capillaries), with local extravasation of red blood cells. Price et al [2] have shown that polymer microspheres could be driven as much as 200 μm into the parenchyma with this method. The authors calculated that only a small number of capillary ruptures were required to deliver large quantities of the colloidal particles to the muscle. Using the same model of polymer microspheres bound to microbubbles and ultrasound, it has also been demonstrated that the ultrasound pulse interval and microvascular pressure influence the creation of extravasation points and the transport of microspheres to the tissue. Both were greatest when the pulse interval was around 5 seconds, which allows maximal microbubble replenishment within the microcirculation after destruction by the ultrasound pulse [19].

The formation of pores in the membranes of cells as a result of ultrasound-induced microbubble cavitation has been proposed as a mechanism for facilitating the drug deposition. Taniyama et al [7] demonstrated the presence of small holes in the surface of endothelial and vascular smooth muscle cells immediately after transfection of a plasmid DNA by ultrasound-mediated microbubble destruction, using electron microscopic scanning. It was then postulated that these transient holes in the cell surface caused by microbubbles and ultrasound resulted in a rapid translocation of plasmid DNA from outside to cytoplasm. Mukherjee et al [10] demonstrated by electron microscopy of a rat heart performed during application of ultrasound, that disruption or pore formation of the membrane of the endothelial cells occurred with acoustic power of 0.8 to 1.0 W/cm^2^. However, it was a lower intensity of ultrasound (0.6 W/cm^2^) that caused more drug delivery with microbubbles. More recently, voltage clamp techniques were used to obtain real-time measurements of membrane sonoporation in the presence of albumin-coated microbubbles (Optison). Ultrasound increased the transmembrane current as a direct result of membrane resistance due to pore formation [20].

Another important therapeutic property of microbubbles is their increased adherence to damaged vascular endothelium. Albumin-coated microbubbles do not adhere to normally functioning endothelium, but their adherence does occur to activated endothelial cells or to extra-cellular matrix of the disrupted vascular wall, and this interaction could be a marker of endothelial integrity [11]. Because of this characteristic, the delivery of drugs or genes bound to albumin-coated microbubbles could be selectively concentrated at the site of vascular injury in the presence [21] or absence of ultrasound application [22].

### Microbubbles Use for Gene Therapy

The clinical use of viral vectors for gene therapy is limited because viral proteins elicit an immune response within the target tissue [23], and have been shown to cause an intense inflammatory activation of endothelial cells [24]. On the other hand, the nonviral delivery of vehicles, such as plasmids and antisense oligonucleotides, has been associated with a lower transfection efficiency and transient expression of the gene product [25]. The first published report of targeted DNA delivery was performed in 1996, using surface ultrasound and intravenously delivered microbubbles carrying antisense oligonucleotides [3]. In 1997, Bao et al [26] described the use of ultrasound and albumin-coated microbubbles to enhance the transfection of luciferase reporter plasmid in cultured hamster cells. Since then, many studies have confirmed the efficacy of ultrasound-mediated microbubble destruction for drug and gene delivery, both in vitro and in vivo [3,7-9]. Shohet et al [9] demonstrated for the first time with an adenovirus vector that the ultrasound-mediated disruption of gas-filled microbubbles could be used to direct transgene expression to the heart in vivo. They showed that intravenously injected recombinant adenovirus vectors encoding a beta-galactosidase reporter gene were successfully delivered to normal rat myocardium using microbubbles and transthoracic 1.3 MHz diagnostic ultrasound, at a mechanical index of 1.5, delivered at a burst of 3 frames of ultrasound every 4 to 6 cardiac cycles. Of note, transfection was not observed if the adenovirus was administered in the same dose without microbubbles, or if the adenovirus was administered with microbubbles but in the absence of ultrasound. Importantly, using the same model the authors confirmed that plasmid transgene expression can be directed to the heart, with an even higher specificity than viral vectors, and that this expression can be regulated by repeated treatments [27].

Taniyama et al [7] have also shown effective transfection of a plasmid DNA to endothelial and vascular smooth muscle cells with albumin-coated microbubbles (Optison) and ultrasound. In vivo studies demonstrated that transfection of wild-type p53 plasmid DNA into balloon-injured blood vessels was effective and resulted in significant inhibition of the ratio of neointimal-to-medial area, as compared with transfection of control vector. In contrast, transfection of p53 plasmid DNA by means of ultrasound without microbubbles failed to inhibit neointimal formation in the rat carotid [7]. In a recent study, Teupe et al [28] have documented efficient transfer of plasmids encoding either beta-galactosidase or endothelial nitric oxide synthase to the endothelial cells of conductance arteries with preservation of the functional integrity of the transfected endothelial cell layer after ultrasound treatment.

### Other Potential Therapeutic Applications of Microbubble Target Drug Delivery

Restenosis after vascular balloon injury or stent deployment has been shown to result from neointimal hyperplasia due to smooth muscle cell migration and proliferation. The c-*myc *protooncogene is responsible for the regulation of gene expression involved in the process of intimal hyperplasia that leads to restenosis. Synthetic antisense oligonucleotides, such as those to the c-*myc *protooncogene, can bind to the messenger ribonucleic acid and inhibit the synthesis of the protooncogenes. Therefore, antisense to c-*myc *protooncogene can prevent its translation into proteins that may be mediators of the pathologic process of restenosis. These synthetic agents, when administered directly into the vessel, have successfully inhibited restenosis after coronary or carotid injury [29]. In 1996 Porter et al [3] demonstrated that perfluorocarbon-exposed sonicated dextrose albumin (PESDA) microbubbles, unlike room air-containing sonicated dextrose albumin microbubbles, have bioactive albumin on their surface that can avidly bind synthetic antisense oligonucleotides, and then release them in the presence of ultrasound. In the initial study that examined the effectiveness of PESDA and ultrasound in enhancing the delivery of the antisense to c-*myc*, 21 pigs had carotid balloon injury performed with an oversized balloon catheter and were randomized to receive intravenous antisense to c-*myc *bound to PESDA, intravenous antisense alone, or no treatment. The pigs that received antisense bound to PESDA also had transcutaneous 20 kHz ultrasound applied over the carotid wall following injections. The ultrasound targeted group showed a significantly lower percent area stenosis (8 ± 2%) than the two control groups (19 ± 8% and 28 ± 3%; p < 0.01) [21].

**Figure 2:**
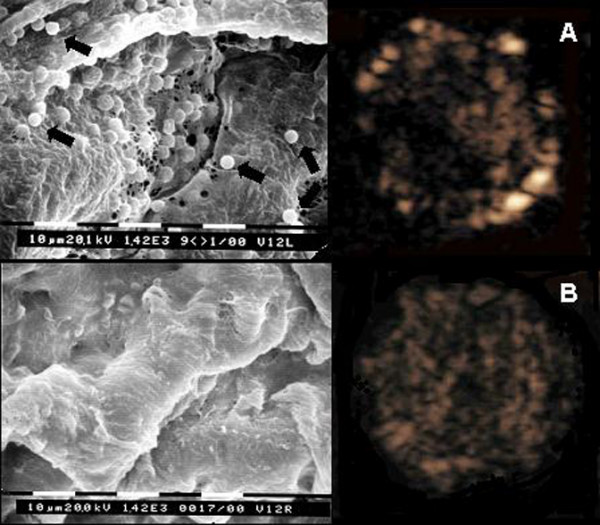
Ultrasound images with low mechanical index pulse sequence scheme showing the presence of microbubbles binding to the arterial endothelium in a balloon-injured carotid artery (Panel **A**, right) and the absence of microbubbles in the control noninjured carotid artery (Panel **B**, right). Scanning electron microscopy (Bar = 10 μm; magnification 1420 ×) revealed sites of injury with endothelial denudation and attachment of microbubbles (black arrows) to the denuded endothelium only in the injured vessel **(A) **and normal appearing endothelium in the control vessel **(B)**. (Reprinted with permission from Tsutsui JM, Xie F, Radio SJ, Phillips P, Chomas J, Lof J et al. **Non-invasive detection of carotid artery endothelial dysfunction due to hypertriglyceridemia and balloon injury with high frequency real time low mechanical index imaging of retained microbubbles**. J Am Coll Cardiol 2004;**44**:1036-46).

Since PESDA microbubbles adhere to sites of endothelial injury even in the absence of ultrasound, the efficacy of this therapy in inhibiting coronary restenosis has been evaluated in animals. Porter et al [22] measured the uptake of antisense to c-*myc *into coronary arteries using high phase liquid chromatography in pigs. Intravenous PESDA containing anti c-*myc *was given in the presence or absence of transthoracic 1 MHz ultrasound (0.6 W/cm^2^). In this study, the authors demonstrated that anti c-myc can be selectively concentrated within a stretch-injured coronary artery segment when given intravenously bound to PESDA. The decrease in neointimal formation following intravenous injection of anti c-myc with PESDA without ultrasound was similar to that observed with higher doses of the same antisense given directly into the coronary artery using an infiltrator delivery system [30]. The basis for this hypothesis stems from previous observations that albumin-coated microbubbles adhere to activated endothelial cells [11,21,31]. Albumin-coated microbubbles have been observed binding to activated leukocytes and monocytes which slowly roll along injured venular endothelial cells [11]. Since leukocyte and monocyte accumulation has also been observed early following arterial balloon injury [32], it is possible that PESDA microbubbles were concentrated at the injured coronary artery surface by adherence to these activated cells. Other potential mechanisms could be related to complement activation, since both albumin- and lipid-encapsulated microbubbles take up complement proteins [33], and thus may bind to upregulated complement receptors at the injured surface. It was recently demonstrated that albumin-coated microbubbles adhere to sites of arterial endothelial dysfunction induced by balloon-injury of carotid arteries [34]. Figure 2 illustrates an example of microbubble binding to the endothelium of an injured carotid artery, which was confirmed by scanning electron microscopy. Lu et al [35] have also shown that albumin-coated microbubbles significantly improved transgene expression in skeletal muscle of mice, even in the absence of ultrasound. However, in this study, the delivery was an intramuscular injection of microbubbles and plasmid into otherwise normal tissue, and not in the setting of endothelial injury [35].

Another innovative application of microbubbles and ultrasound is in the delivery of proteins that induce growth of endothelial cells, such as vascular endothelial growth factor (VEGF). Mukherjee et al [10] demonstrated a marked increase in endothelial VEGF uptake using ultrasound alone (eight-fold increase) and using ultrasound and PESDA (ten- to thirteen-fold increase, as compared to control) in the myocardium of rats. In a canine model of chronic myocardial ischemia, intravenous infusion of VEGF combined with ultrasound and an albumin-based contrast agent significantly reduced the infarct area/risk area ratio, and increased myocardial blood flow in the ischemic territory, suggesting a new potential therapeutic approach for angiogenesis [36].

### Optimization of Ultrasound Parameters for Cardiac Drug and Gene Delivery

The effect of several ultrasound parameters, including transducer frequency and acoustic power, are known to influence microbubble destruction and, thus, the transfection of genes and drugs. Although the optimal ultrasound parameters for maximizing this process are not known, we will briefly discuss some important aspects. Unger et al [6] have shown that the type of ultrasound used to destroy phospholipid-coated microbubbles may regulate how much drug is released in vitro. When analyzing the number of acoustically active particles that persist after exposure to different types of ultrasound in a flow chamber, they demonstrated that a 2.5 MHz transducer resulted in some destruction, but the addition of a lower-frequency transducer (100 kHz) significantly increased the destruction. When the 100 kHz energy was given in a pulsed-wave mode as opposed to a continuous wave, the destruction of microbubbles was even faster. In a similar way, Porter et al [21] have demonstrated that a continuous wave diagnostic ultrasound frequency of 2 MHz was not able to enhance the carotid uptake of antisense to c-*myc *protooncogene (0.19 ± 0.04 μg/mg), but low-frequency 20 kHz ultrasound significantly increased vascular uptake (0.28 ± 0.04 μg/mg; p = 0.008 vs other groups) when compared to antisense bound to PESDA alone (0.21 ± 0.06 μg/mg). The results of this study suggested that a lower frequency could be better suited to target antisense deposition into major vessels. Because there were minimal differences in peak negative pressure generated by 2 MHz and 20 kHz in this study (46 kPa and 13 kPa, respectively), the enhanced uptake was attributed to a lower threshold for cavitation with 20 kHz ultrasound frequency.

In another study, the efficacy of ultrasound-mediated delivery of VEGF bound to PESDA into the myocardium of rats was evaluated with an ultrasound frequency of 1.0 MHz at various acoustical outputs (0.2, 0.4, 0.6, 0.8 and 1.0 W/cm^2^). The authors found a significant increase in VEGF uptake with the combination of ultrasound and PESDA at all power outputs when compared with controls, but there was a dose-dependent increase in the amount of VEGF uptake with increasing power until 0.6 W/cm^2 ^and a subsequent plateau. Table 1 illustrates some parameters used in previous studies for drug and gene delivery. It seems that at higher frequencies, higher peak negative pressures are necessary to induce cavitation-mediated drug and gene delivery using microbubbles and ultrasound. In a recent study of Chen et al [8] it was shown that, when using ultrasound at diagnostic frequencies, optimal ultrasound parameters for gene expression by ultrasound-targeted microbubble destruction to the myocardial microcirculation included a low-transmission frequency (1.3 MHz), high mechanical index, and electrocardiogram triggering to allow complete filling of the myocardial capillary bed by microbubbles. The authors found that maximal acoustic pressure resulted in higher myocardial gene expression, providing indirect evidence that high peak negative pressures increase the amount of gene delivery from microbubbles. Furthermore, the optimal ultrasound parameters for targeted delivery may be dependent on the desired site for delivery. While a triggered mechanism of once every four to five seconds may work for delivering drugs by ultrasound-mediated destruction of microbubbles in the myocardial microcirculation, a more frequent pulsed delivery may be required for vascular delivery.

**Table 1:** Ultrasound parameters and microbubbles used for delivering genes and drugs.

**Author**	**Transfection**	**Transducer frequency**	**Delivery mode**	**Delivery site**	**Output**	**Peak negative pressure**	**Microbubble**	**Efficacy**
Porter TR, et al^1^	Antisense c-*myc *protooncogene	1 MHz	PW	Coronary arteries	0.6 W/cm^2^		PESDA	+
Zhou Z, et al^2^	VEGF	1 MHz	CW	Myocardium	1.2 W/cm^2^		Sonazoid	+
Taniyama Y, et al^3^	Luciferase			Carotid artery	2.5 W/cm^2^		Optison	+
Teupe C, et al^4^	β-galactosidase/ eNOS	2.2–4.4 MHz	CW	Coronary arteries			Gas-filled albumin microbubble	+
Porter TR, et al^5^	Antisense c-*myc *protooncogene	2 MHz	CW	Carotid artery		13 kPa	PESDA	-
		20 kHz	CW	Carotid artery		46 kPa	PESDA	+
Mukherjee D, et al^6^	VEGF	1.0 MHz	CW	Myocardium	0.2 W/cm^2^	0.164 MPa	PESDA	9.37 ± 1.98*
		1.0 MHz	CW	Myocardium	0.4 W/cm^2^	0.194 MPa	PESDA	18.58 ± 2.46*
		1.0 MHz	CW	Myocardium	0.6 W/cm^2^	0.328 MPa	PESDA	23.12 ± 3.95*
		1.0 MHz	CW	Myocardium	0.8 W/cm^2^	0.394 MPa	PESDA	25.46 ± 2.78*
		1.0 MHz	CW	Myocardium	1.0 W/cm^2^	0.419 MPa	PESDA	26.48 ± 3.98*
Shohet RV, et al^7^	β-galactosidase	1.3 MHz	ECG-triggered	Myocardium			Perfluorocarbon-filled microbubbles	+
Bao S, et al^8^	Luciferase	2.25 MHz		Cultured cells		0.2–0.4 MPa	Albunex	+

* Efficacy is demonstrated as mean ± SD endothelial vascular growth factor uptake by enzyme-linked immunosorbent assay. CW = continuous wave; ECG = electrocardiogram; eNOS = endothelial nitric oxide synthase; PESDA = perfluorocarbon-exposed sonicated dextrose and albumin; PW = pulsed wave; VEGF = vascular endothelial growth factor.

However, a high peak negative pressure may have detrimental bioeffects. Several investigators have reported on the occurrence of tissue hemorrhage and endothelial cell damage after ultrasound exposure of cultured cells and organs containing air, such as the lungs or the intestine [37-39]. Ay et al [38] examined the functional and morphological effects of ultrasound and contrast in an isolated rabbit heart preparation, using increasing levels of acoustic energy. Simultaneous exposure to contrast and high-energy ultrasound resulted in a reversible and transient decrease in left ventricular contractile performance, increase in the coronary perfusion pressure, increase in the myocardial lactate release, and presence intramural hemorrhage in the plane of ultrasound transmission. Additionally, light microscopy revealed the presence of capillary ruptures, erythrocyte extravasation and endothelial cell damage. These effects were directly related to the mechanical index. These studies indicate that although high-energy ultrasound seems to be necessary to induce tissue permeability facilitating local drug delivery, it may also have significant bioeffects in the myocardium. Therefore, the optimal ultrasound parameters to enhance drug delivery with microbubbles remain to be determined.

## Competing interests

Dr. Jeane M. Tsutsui – declares no competing interests.

Dr. Feng Xie – declares no competing interests.

Dr. Thomas R. Porter – declares ImaRx Therapeutics, Inc.: Grant support and Consultant; Bristol Myers Squibb Medical Imaging: Grant support; AVI BioPharma, Inc.: Grant support

**Figure 1:**
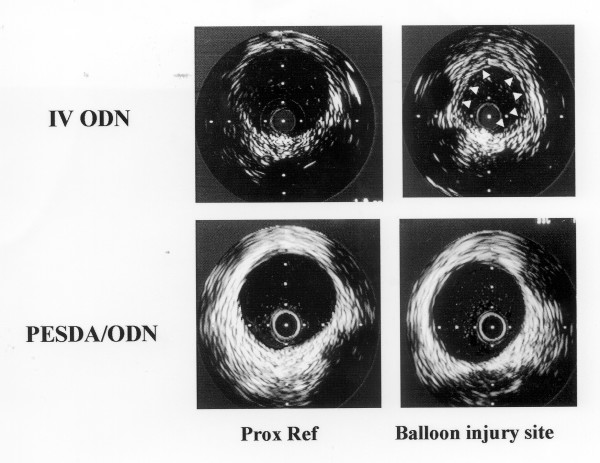
Intravascular ultrasound examples of the proximal reference site and balloon injury site 30 days after the angioplasty. Note that there was both greater intimal thickening (arrows) in the vessel treated with intravenous antisense alone, and a reduction in lumen size when compared to the proximal reference segment. The balloon injury site of the vessel treated with intravenous antisense plus PESDA and 20 kHz transcutaneous ultrasound did not exhibit any reduction in lumen area or visually evident plaque. (Reprinted with permission from Porter TR, Hiser WL, Kricsfeld D, Deligonul U, Xie F, Iversen P et al: **Inhibition of carotid artery neointimal formation with intravenous microbubbles**. Ultrasound Med Biol 2001, **27**:259-265).
